# Prospective Case Study of Outcome of Tibial Plateau Fractures Treated with Locking Condylar Plate

**DOI:** 10.5704/MOJ.1611.007

**Published:** 2016-11

**Authors:** R Jain

**Affiliations:** Department of Orthopaedics, Sanjay Gandhi Memorial Hospital, New Delhi, India

## Abstract

**Introduction:** Tibial plateau injury involves the articular surface of the proximal tibia with diverse group of fractures that represent a wide spectrum of severity that challenge even the most experienced surgeons, but with the invent of modern diagnostic technology and the advent of locking plates, results appear to be improving over time.

**Method:** The study was conducted on thirty patients admitted in the department of orthopaedics with diagnoses of tibial plateau fractures treated with Locking Condylar Plate. The patients were followed up for a minimum period of six months and clinico-radiological progression of fracture union with the functional outcome was studied using 100 point rating system devised by Delamarter *et al*.

**Result:** Twenty-two patients showed excellent results while eight patients had good to fair and none with poor result. The average time for radiological union was 17.5 weeks.

**Conclusion:** The results of the study concluded that while locking condylar plate seems to show excellent results in low energy tibial plateau fractures, it can probably be used to successfully treat patients with high energy fracture patterns without the need for additional medial stabilization.

## Introduction

Fractures of the tibial plateau were first described as car bumper fractures^1^. Tibial plateau fractures are a diverse group of fractures that represent a wide spectrum of severity which ranges from simple injuries to complex fracture patterns that challenge even the most experienced surgeons^2^.

Conservative and closed treatment of displaced tibial plateau fractures resulted in unacceptably high rates of joint stiffness, malunion and osteoarthritic changes^3^. With the advances in internal fixation principles, operative treatment modalities were explored; however they were often associated with high complication rates^4^. With modern diagnostic technology, less invasive surgical approaches and the advent of locking plates, results appear to be improving over time^5^. Laterally-based locking plates provide increased stability in the presence of metaphyseal or metadiaphyseal comminution, and may offer an alternative to an additional medial plate or external fixator for added support of the medial column when a non-locking plate implant was used for bicondylar fractures^6^.

The biomechanical advantages of locking plate constructs are not easily realized when used in these relatively straightforward fracture patterns. Yet the enthusiasm for use of these expensive devices in even the simplest of fractures has become common^7,8^.

The introduction of locking plates for treatment of complex tibial plateau fractures holds many potential advantages, including increased holding power in osteopenic bone, unicortical purchase in periarticular region and ability to successfully and stably bridge severely comminuted meta diaphyseal shaft areas^9,10^.

The present study was undertaken to evaluate the anatomical union and functional outcome of fixation of tibial plateau fractures with locking condylar plate.

## Materials and Methods

This prospective study included thirty patients above 18 years of age with acute unilateral tibial plateau fractures, admitted to the department of orthopaedics. Though patients with associated upper limb injuries were included, patients with associated head and abdominal injury, other lower limb and floating knee injuries or Gustilo Anderson grade III C with neurovascular injuries were excluded from the study.

**Fig. 1:**
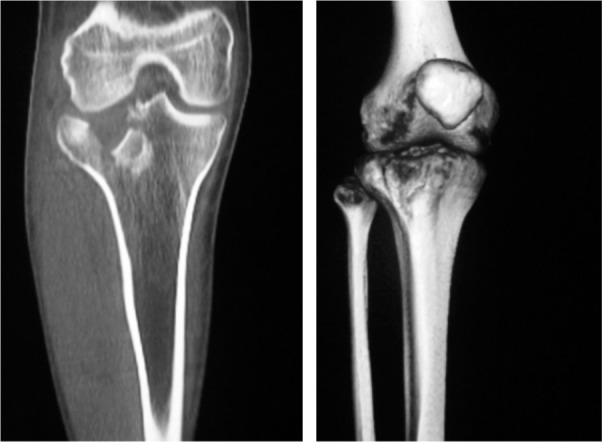
Pre-operative three-dimensional reconstruction CT scan showing Schatzker type II split depression fracture.

Fractures were classified according to the Schatzker classification using plain radiographs or three dimensional reconstruction CT scans (Fig. 1) when required. Patients were positioned supine on a radiolucent table and image intensifier (C-arm) was kept ready to be used intraoperatively. Using anterolateral approach, a curvilinear incision was made over the proximal lateral tibia. The fascia of the iliotibial band was divided longitudinally parallel to its fibers starting at the Gerdy tubercle and extending proximally, and distally the dissection was extended through the fascia of the tibialis anterior muscle. A small portion of the muscle was elevated from the proximal lateral tibia and the articular surface was restored, fracture reduced, checked with an image intensifier and bone fragments were secured with K-wire. Appropriate size precontoured lateral locking plate with at least four screws in distal cortex was selected and inserted between the tibialis anterior and the periosteum. The plate position and fracture alignment was confirmed with image intensifier in both planes and fixation was done using locking screws in the head of tibial plate and either locking or non-locking screws were used distally to pull bone to plate, as and when required. In case of depression, the depressed articular fragment and compressed cancellous bone were elevated and filled with bone graft. The wound was closed in layers to cover the implants and bone.

**Fig. 2:**
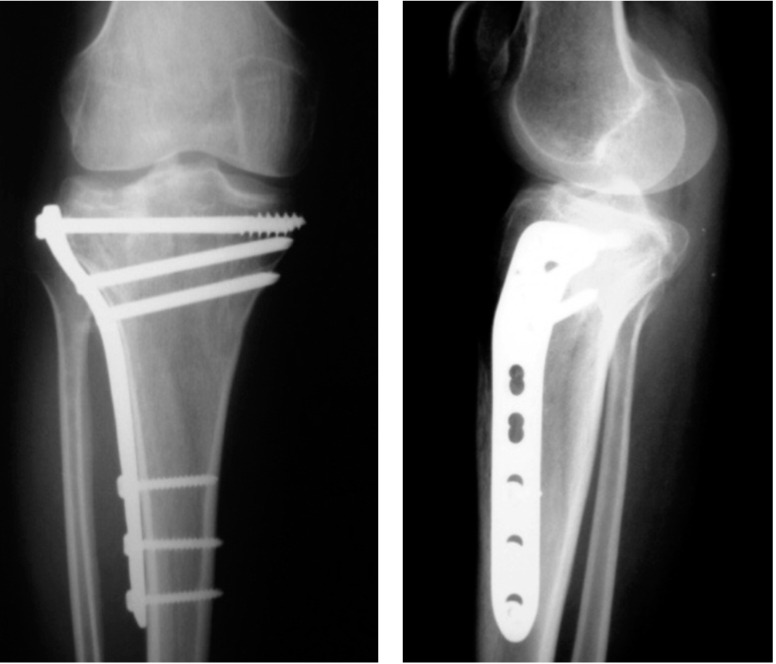
Follow up radiograph at 6 months showing healed fracture and a preserved joint with alignment well maintained.

Postoperatively patients were put in a long knee brace for two weeks. Isometric quadriceps exercises and knee range of motion were encouraged from the third day. Mobilization was started as soon as pain permitted; first with non-weight bearing, crutch support walking, followed by toe-touch crutch support walking and then progressive weight bearing depending upon tolerance and radiographic evidence of fracture healing. Follow up visits were at six weeks’ interval until fracture healing was seen and at follow up visits the fractures were evaluated clinically and radiologically for healing and alignment (Fig. 2). Final result were assessed using a 100-point rating system devised by Delamarter *et al*(Table I) and were graded as excellent (90-100 points), good (80-89 points), fair (70-79 points) or poor (< 70 points) ^11,12^.

**Table I:** 100-point rating system devised by Delamarter *et al*11

SYMPTOMATIC (MAXIMUM OF 30 POINTS)			FUNCTIONAL (MAXIMUM OF 45 POINTS)			ANATOMIC(MAXIMUM OF 25 POINTS)		
1.	Pain (maximum of 20 points)	Points	1.	Knee Flexion (Maximum of 30 points)	Points	1.	Angular deformity (Maximum of 10 points)	Points
	None	20		>135°	30		No deformity	10
	Wet weather ache	18		120-134°	26		5°	08
	After hard use	16		105-119°	23		10°	05
	Limits use	14		90-104°	20		15°	02
	Painful walking	10		75-89°	15		20 ° or more	00
	Limits walking	05		60-74°	10			
	Continuous pain	00		<60°	05			
				Ankylosed	00			
2.	Activity level		2.	Extension		2.	Instability	
	(maximum of 5 points)			(Maximum of 10 points)			(Maximum of 10 points)	
	Unlimited	05		Full Extension	10		No instability	10
	Limited in sports	04		Lacks 1-5°	05		5°	08
	Limited in jogging	03		Lacks 6-10°	02		10°	05
	Limited in walking	02		Lacks more than 10°	00		15°	02
	Cane/Brace needed	00					20° or more	00
3.	Patient Assessment		3.	Crepitus		3.	Arthritis	
	(maximum of 5 points)			(Maximum of 5 Points)			(Maximum of 5 points)	
	Normal knee (100%)	05		No crepitus	05		None	05
	Near normal (90%)	04		Clicking	04		Mild	04
	Good (>75%)	03		Occasional locking	03		Moderate	02
	Fair (50-75%)	01		Constant Crepitus	01		Severe	00
	Poor (<50%)	00						

Union was defined as pain-free full weight bearing in the absence of tenderness or movement at the fracture site with the presence of bridging callus across at least one cortex of fracture site on each of the anteroposterior and lateral radiological views^13^. Non-union was defined as absence of progressive fracture healing for three consecutive months extending beyond six months from date of injury^13^ and malunion was defined as step-off of the articular surface of greater than 2 mm on anteroposterior and lateral knee radiographs or malalignment of greater than 5 degree in any plane on full length tibia. A significant loss of knee range of motion was defined as flexion <90 degree^4^.

The observation and results obtained were subjected to standard statistical analysis which included mean, median, mode and standard deviation using SPSS16 software. Test used to calculate p-value was χ^2^ (Chi-Square) test. Depending upon the p-value obtained by the above mentioned test, results were interpreted as significant if p value was <0.05.

## Result

**Fig. 3:**
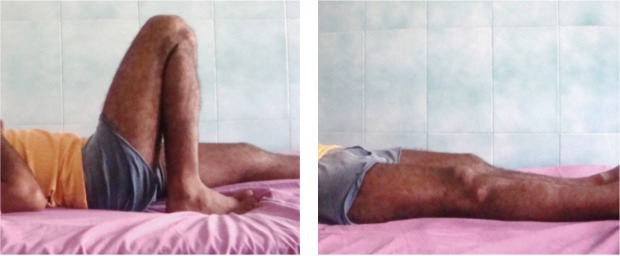
Clinical photographs showing range of movement at knee with flexion more than 135 degrees and full extension.

The present prospective study comprised 30 patients with tibial plateau fractures with an average age of 50.96 years (ranging from 28 to 76 years) with male to female ratio of 5:1 with majority of fractures being simple fractures (63.3%). As per Schatzker classification (Table II), type II and type III each constituted 23.3% with type V (6.7%) and nine-hole locking condylar plate was most commonly used (53.3%). While four patients required an additional interfragmentary screw, eight patients of type II and type III required primary bone grafting. Post-operatively knee mobilization was started at an average interval of 5.27 days with mean time of 9.8 weeks for partial weight bearing and 16.3 weeks for full weight bearing. The average time for radiological union (Table III) was 17.5 weeks (14.5 weeks for type I, 15.4 weeks for type II, 16.3 weeks for type III, 17.5 weeks for type IV, 21.0 weeks for type V, and 22.3 weeks for type VI) following which 19% patients had flexion more than 135 degree while 26 % had full extension (Fig. 3). Five patients (16.6%) with infection were adequately treated with antibiotics and three patients with lateral meniscus tear, out of which one patient with associated ACL tear were managed conservatively. The post-operative malalignment occurred in nine patients and articular malreduction in five patients with clinical significance of more than 5 degree and more than 2 mm depression or split respectively in two cases each and since both had no complaint apart from pain, they were managed conservatively. Three patients had problem with hardware in the form of either consistent pain or screw irritation on medial side and in two of these patients hardware was removed once the union was achieved and the other patient was managed conservatively.

**Table II:** Schatzker Type

Schatzker type	No. of patients	Percentage (%)
I	4	13.3
II	7	23.3
III	7	23.3
IV	4	13.3
V	2	6.7
VI	6	20.0

**Table III:** Radiological Union versus Schatzker Type

Radiological Union				Schatzker Type			Patients	Percentage (%)
(weeks)	I	II	III	IV	V	VI		
14-16	4	5	4	2	-	-	15	50.0
17-19	-	2	2	1	1	1	7	23.3
20-22	-	-	1	1	-	3	5	16.7
23-25	-	-	-	-	1	1	2	6.7
26-28	-	-	-	-	-	1	1	3.3
χ2, df, p-value	26.898,	20,0.138						

According to Chi-square test in the present series (Table IV), it was found that results showed significant statistical association (p<.05) with the Schatzker fracture type. High energy tibial plateau fractures (62.5% of type V and VI) tended to show less favourable, though still good results compared to monocondylar fractures. Excellent results were seen in 73.3% patients while eight patients with associated factors like severe injury in terms of fracture pattern, other associated injuries, open fractures, etc., had good to fair results. There was no patient with poor result.

**Table IV:** Results versus Schatzker Type

Results				Schatzker Type			No. of Patients	Percentage (%)
	I	II	III	IV	V	VI		
Excellent	4	7	7	3	-	1	22	73.3
Good	-	-	-	1	1	3	5	16.7
Fair	-	-	-	-	1	2	3	10.0
Poor	-	-	-	-	-	-	-	-
Total patients	4	7	7	4	2	6	30	100.0
χ2, df, p value	23.008, 10,	0.011*						

(χ^2^ Chi square, df degree of freedom, * significant)

## Discussions

Tibial plateau fractures occur more commonly in the third to fifth decade of life with the mean age of 50.9 years in the present study (n=30), comparable to studies done by Mahajan *et al*^12^ (n=25) and Ricci *et al*^13^ (n=38). Unicondylar fractures were most commonly seen with 22 patients in the present study and the average time for partial and full weight bearing was 9.8 weeks and 16.3 weeks respectively which was comparable to study done by Mahajan *et al*^12^. In the present study it was observed that the Schatzker type had no significant association (p=0.138) with radiological union with the average time to union of 17.5 weeks which was comparable to studies by Mahajan *et al*^12^ and Stannard *et al*^14^. However other factors like open fracture at the time of injury, post-operative infections, smoking habits, and others, do affect the time to union. In the present study five patients had infection which was comparable to studies by Gosling *et al*^15^ and Phisitkul *et al*^16^ but the varying criteria used for postoperative malalignment in different series, the quality of radiographs and the reliability of the measurement techniques make it hard to compare with other series.

The results were comparable with study of Mahajan *et al*^12^ which had 92 % of good to excellent results. Delamarter *et al*^11^ in their study of 39 patients found 28.20% to have poor results. This was due to the fact that all the patients selected in their study either had isolated ligament injury or combined ligament injuries associated with tibial plateau fracture which resulted in instability. Fair results in the present study had shown strong association with open injuries which developed infections, severe injuries in term of fracture pattern (type V and VI p=0.011) and associated ligaments and meniscal injuries.

Our study has several limitations. The number of patients in our study and follow up period is a weakness. Long term follow up may reveal patients showing post-traumatic arthritic changes which would certainly lead to additional outcomes.

## Conclusion

We conclude that locking condylar plate provides a fixed angle construct secondary to the locking screw and plate design which creates an implant that seems to show excellent functional and anatomical results for low energy fractures with involvement of either of the condyle. It probably can also be used successfully to treat patients with high energy fracture patterns without the need for additional medial stabilization as it provides restoration of the articular surface with better biomechanical stability, increased range of motion, decreased complications like infections, non-union and early rehabilitation.
